# Supplementary material for: Putting situational affordances in an intervention context: How the interaction between personality and intervention situations can help us explain differential intervention responses
Source: PLoS One. 2024 Dec 17;19(12):e0309180. doi: 10.1371/journal.pone.0309180 (PMC11651624; doi:10.1371/journal.pone.0309180)
Supplement: S1 File — (DOCX) [file pone.0309180.s001.docx]

Putting Situational Affordances in an Intervention Context: How the interaction between personality and intervention situations can help us explain differential intervention responses

Esther C.A. Mertens^1^, Isabel Thielmann^2^, Annalaura Nocentini^3^, Aniek M. Siezenga^1,2^, & Jean-Louis van Gelder^1,2*^

^1^ Institute of Education and Child Studies, Leiden University, the Netherlands

^2^ Department of Criminology, Max Planck Institute for the Study of Crime, Security and Law, Germany

^3^ Department of Educational Sciences and Psychology, University of Florence, Italy

**Supporting information**

**Participants: COVID-19 lockdown**

During data collection there was a lockdown due to the COVID-19 pandemic. We compared participants who were included before the lockdown (n = 119) with participants included after the lockdown (n = 57) and found no significant differences in age, gender, vividness of future self, valence towards future self, self-defeating behaviors, psychosocial wellbeing, self-esteem, self-efficacy, and future orientation. However, there was a significant difference, albeit of small relevance, between the groups on relatedness towards the future self (*F*(1,174) = 4.14, *p* = .043, η^2^_partial_ = .023), with participants reporting more relatedness after the lockdown than before. Given that we found only one small difference between participants before and after the lockdown, we did not control for the lockdown in the analyses.

**S1 Table. Descriptive statistics of the outcome variables at each measurement occasion and of personality traits per condition.**

|  | |  | **Intervention condition (*n* = 87)** | | | |  | **Control condition (*n* = 89)** | | | |
| --- | --- | --- | --- | --- | --- | --- | --- | --- | --- | --- | --- |
|  | |  | T1 | T2 | T3 | T4 |  | T1 | T2 | T3 | T4 |
|  | |  | *M* (*SD*) | *M* (*SD*) | *M* (*SD*) | *M* (*SD*) |  | *M* (*SD*) | *M* (*SD*) | *M* (*SD*) | *M* (*SD*) |
| **Future self-identification** | | |  |  |  |  |  |  |  |  |  |
|  | **Vividness** | | 3.24 (1.49) | 3.83 (1.31) | 3.95 (1.42) | 3.83 (1.45) |  | 3.50 (1.42) | 3.77 (1.34) | 3.82 (1.38) | 3.76 (1.36) |
|  | **Valence** | | 6.59 (1.62) | 6.49 (1.29) | 6.39 (1.29) | 6.45 (1.35) |  | 6.73 (1.14) | 6.61 (1.26) | 6.51 (1.14) | 6.59 (1.18) |
|  | **Relatedness** | | 3.76 (1.11) | 3.83 (1.11) | 4.05 (1.07) | 4.16 (1.07) |  | 3.94 (1.03) | 3.95 (1.06) | 4.08 (0.99) | 4.20 (1.10) |
| **Personality traits** | | |  |  |  |  |  |  |  |  |  |
|  | **Conscientiousness** | | 3.46 (0.63) |  |  |  |  | 3.56 (0.60) |  |  |  |
|  | **Openness to Experience** | | 3.21 (0.59) |  |  |  |  | 3.11 (0.62) |  |  |  |
|  | **Extraversion** | | 3.46 (0.57) |  |  |  |  | 3.48 (0.52) |  |  |  |
|  | **Honesty-Humility** | | 3.51 (0.61) |  |  |  |  | 3.70 (0.48) |  |  |  |
|  | **Emotionality** | | 3.30 (0.61) |  |  |  |  | 3.52 (0.49) |  |  |  |
|  | **Agreeableness** | | 3.01 (0.62) |  |  |  |  | 3.24 (0.51) |  |  |  |

T1 = Baseline; T2 and T3 = Interim measurements; T4 = Post measurement.

**Linear mixed models including all interactions**

To test whether significance of the three-way interactions were driven by significant underlying two-way interactions, we also analyzed models in which we included all possible two-way interactions of the three-way interactions. The results of these models showed the same patterns as the models only including the three-way interactions of interest, indicating the robustness of our findings (see S2 Table and S3 Table).

**S2 Table. Results of the linear mixed models regarding Conscientiousness, Openness to experience, and Extraversion per outcome including all two-way interactions.**

|  | **Vividness** | | | | **Valence** | | | | | **Relatedness** | | |
| --- | --- | --- | --- | --- | --- | --- | --- | --- | --- | --- | --- | --- |
|  | *F* | | *p* |  | | *F* | | *p* |  | *F* | | *p* |
| **Time** | 22.72^**^ | < .001 | | 2.04 | | | .107 | | 12.58^**^ | | < .001 | |
| **Gender** | 2.99 | .085 | | 0.61 | | | .435 | | 1.13 | | .290 | |
| **Condition** | 0.00 | .969 | | 0.59 | | | .445 | | 0.30 | | .587 | |
| **Conscientiousness** | 8.61^**^ | .004 | | 5.72^*^ | | | .018 | | 6.63^*^ | | .011 | |
| **Openness** | 0.33 | .566 | | 0.29 | | | .594 | | 0.01 | | .932 | |
| **Extraversion** | 11.46^**^ | .001 | | 51.65^**^ | | | < .001 | | 15.77^**^ | | < .001 | |
| **Time*condition** | 2.77^*^ | .041 | | 0.03 | | | .993 | | 0.28 | | .842 | |
| **Time*Conscientiousness** | 0.87 | .456 | | 1.66 | | | .175 | | 0.31 | | .816 | |
| **Condition*Conscientiousness** | 0.85 | .357 | | 0.00 | | | .964 | | 1.20 | | .275 | |
| **Time*Openness** | 0.24 | .872 | | 0.07 | | | .976 | | 1.07 | | .361 | |
| **Condition*Openness** | 0.30 | .583 | | 0.84 | | | .361 | | 0.86 | | .356 | |
| **Time*Extraversion** | 1.13 | .338 | | 2.36 | | | .071 | | 0.38 | | .767 | |
| **Condition*Extraversion** | 0.01 | .910 | | 2.43 | | | .121 | | 0.10 | | .756 | |
| **Time*condition*Conscientiousness** | 0.31 | .816 | | 0.75 | | | .522 | | 0.53 | | .662 | |
| **Time*condition*Openness** | 2.37 | .070 | | 1.45 | | | .227 | | 0.97 | | .404 | |
| **Time*condition*Extraversion** | 0.67 | .571 | | 2.31 | | | .075 | | 1.39 | | .246 | |

* p < .05

** p < .01

**S3 Table. Results of the linear mixed models regarding Honesty-Humility, Emotionality, and Agreeableness per outcome including all two-way interactions.**

|  | **Vividness** | | | | **Valence** | | | | | **Relatedness** | | | |
| --- | --- | --- | --- | --- | --- | --- | --- | --- | --- | --- | --- | --- | --- |
|  | *F* | | *p* |  | | *F* | *p* | |  | | *F* | | *p* |
| **Time** | 23.41^**^ | < .001 | | 1.71 | | | .164 | 12.75^**^ | | | | < .001 | |
| **Gender** | 2.14 | .145 | | 1.30 | | | .256 | 0.49 | | | | .483 | |
| **Condition** | 0.24 | .622 | | 0.92 | | | .338 | 0.84 | | | | .362 | |
| **Conscientiousness** | 10.56^**^ | .001 | | 6.22^*^ | | | .014 | 1.23 | | | | .270 | |
| **Openness** | 0.30 | .586 | | 0.13 | | | .722 | 6.39^*^ | | | | .012 | |
| **Extraversion** | 12.03^**^ | .001 | | 53.17^**^ | | | < .001 | 17.91^**^ | | | | < .001 | |
| **Honesty-Humility** | 0.16 | .693 | | 0.43 | | | .513 | 0.70 | | | | .404 | |
| **Emotionality** | 1.81 | .180 | | 0.53 | | | .468 | 7.56^**^ | | | | .007 | |
| **Agreeableness** | 1.59 | .209 | | 0.42 | | | .518 | 0.01 | | | | .909 | |
| **Time*Condition** | 3.48^*^ | .016 | | 0.07 | | | .974 | 0.37 | | | | .772 | |
| **Time*HH** | 1.37 | .251 | | 0.63 | | | .597 | 0.23 | | | | .874 | |
| **Condition*HH** | 0.68 | .410 | | 2.71 | | | .101 | 1.21 | | | | .274 | |
| **Time*Emotionality** | 1.45 | .228 | | 1.22 | | | .303 | 2.78^*^ | | | | .041 | |
| **Condition*Emotionality** | 0.65 | .422 | | 0.03 | | | .853 | 0.01 | | | | .938 | |
| **Time*Agreeableness** | 3.24^*^ | .022 | | 0.20 | | | .896 | 0.31 | | | | .818 | |
| **Condition*Agreeableness** | 2.40 | .123 | | 0.11 | | | .745 | 0.04 | | | | .849 | |
| **Time*condition*HH** | 0.10 | .962 | | 0.19 | | | .907 | 0.30 | | | | .829 | |
| **Time*condition*Emotionality** | 0.86 | .464 | | 1.20 | | | .308 | 0.23 | | | | .873 | |
| **Time*condition*Agreeableness** | 1.57 | .197 | | 1.60 | | | .189 | 1.34 | | | | .261 | |

Reported statistics of main effects and covariates are based on the model that included the interaction-effect with Honest-Humility as these effects were highly similar across models.

* *p* < .05

** *p* < .01
