# Supplementary material for: Putting situational affordances in an intervention context: How the interaction between personality and intervention situations can help us explain differential intervention responses
Source: PLoS One. 2024 Dec 17;19(12):e0309180. doi: 10.1371/journal.pone.0309180 (PMC11651624; doi:10.1371/journal.pone.0309180)
Supplement: Attachment — Submitted filename: Appendix Response to Reviewers.docx [file pone.0309180.s002.docx]

**Appendix Response to Reviewers**

**Reviewer #1:**

1. The introduction focuses on the effects of interventions on the expression of individual differences, i.e., that – depending on the intervention – some people may show stronger intervention-related outcomes based on their personality. However, the hypotheses, noted in the section on ‘The present study’ are about the ‘moderating role’ of personality on the intervention – outcome relation, which is something different. That is, the situational affordances framework argues that situations may moderate personality – criteria relations; here it is argued that personality moderates the situation – criteria relations. I know that – technically – this may entail the same analysis, but conceptually it is a different thing. Please make sure that you remain consistent through the manuscript which conceptualization is used.

*Response: We agree and thank the Reviewer for pointing this out. We have adjusted the title and text accordingly throughout the manuscript.*

*For example, “The present study aimed to illuminate the potential of situational affordances for improving our understanding of personality influences in intervention contexts. To this end, we described the FutureU intervention in terms of intervention situations and corresponding affordances and examined whether the intervention situation (compared to the control condition) moderated the relation between personality traits and intervention effects.” (p. 8)*

2. Related to the above, it is unclear whether direct effects of personality and/or the situation were expected. Based on the absence of these direct effects in the preregistration, one would almost assume that no direct effects are hypothesized, but it would be highly unusual to expect pure moderator effects, so some direct effects are to be expected. There is some indication that the researchers expected a direct effect of the intervention in that FutureU ‘aims to strengthen people’s identification with their ‘future self’ in order to increase their future-oriented thinking and behavior’. Maybe it would be good to clearly state what the direct effects are in ‘The present study’ (although the authors might make a footnote in which they note that this wasn’t preregistered). Even less clear in the introduction is whether personality was expected to have a direct effect (which is actually the case in this study). It would be good to also make clear to the reader whether such direct effects were to be expected.

*Response: Following the recommendation of the Reviewer we now explicitly mention the direct effects in ‘The present study’.*

*“Furthermore, we expected a direct, positive effect of the FutureU intervention on future self-identification given that it aims to increase people’s identification with their future self. We had no specific hypotheses regarding direct effects of personality traits on future self-identification.”(p. 9)*

3. Although I appreciate the broad introduction on interventions and affordances, the sudden content shift to the ‘first test of this affordance-based intervention framework’ is large and is hard to follow for an unacquainted reader. I would either like to see – at the very beginning – a starting paragraph which introduces the central question and variables of this study before introducing the theoretical framework, or I would like the authors to make a better connection between the first (intervention and affordances) part and the second (FutureU) part. I prefer the first option. It also would be good to use some headers to help the reader navigate through the introduction because it contains a lot of different information.

*Response: This was a helpful and clarifying comment. We have now added the central aim and the specific variables of the present study in the first paragraph in the introduction. Additionally, we added headers in the introduction to help the reader navigate it.*

*“In the present study, we propose that interventions place individuals in different situations that may afford the expression of different personality traits and, thereby, allow different individuals to benefit more, or less, from that specific intervention. More specifically, we examined whether intervention situations created by the FutureU intervention moderated the relation between individuals’ personality traits and the intervention’s effects on future self-identification.” (p. 3)*

4. Table 1 helps, but I would make sure that it is ordered in terms of the personality traits. Note that the outcome variable of interest is future self-identification; the authors might like to feature this in the table (or its heading) as well.

*Response: We have reordered Table 1 as suggested. As this is a representation of the information provided in the text, we put the focus here on which intervention situations could afford the expression of certain personality traits rather than the outcomes of the intervention itself.*

5. What I miss in the introduction, in Table 1, and in the methods section is how the FutureU intervention differs from the control condition in terms of the situational affordances. This is the most important variable of this study, and it is not clear from the introduction and beyond how the intervention is supposed to afford the expression of these three traits more than the control condition. Please explain. The control condition – or at least the kinds of situations that the control condition offers – might be featured in the Table 1 as well.

*Response: Participants in the intervention condition set goals and received the FutureU intervention, while the control condition only set goals. In terms of intervention situations, individuals in the intervention condition encountered all the intervention situations mentioned in Table 1, whereas individuals in the control condition only encountered an intervention situation affording goal-directed behavior. We now explicitly describe this difference in the introduction and added a note below Table 1 stating that participants in the control condition did not encounter the described intervention situations except for setting goals. Additionally, we now emphasize it again in the method section.*

*For example, “The FutureU intervention situations were not apparent in the control condition, except for setting goals. As in the intervention condition, people in the control condition set goals, creating a situation affording goal-directed behaviors, but in contrast to the intervention condition they received no further intervention and, thus, did not encounter other intervention situations.” (p. 8)*

*“Hence, besides setting weekly goals for three weeks, the situations encountered by the participants in their daily lives were not affected by an intervention.” (p. 12)*

6. Interventions are usually targeted at behaviors, skills, and/or attitudes, and I understand from the introduction that the ultimate goal is to help participants to better set – and adhere – to goals. I also gather from the introduction (although it is not clearly stated as such) that future self-identification is thought to act as a mediator or moderator of the relation between the intervention and the ultimate goal of the intervention (i.e., more (future) goal-directed behaviors). To the reader, it is not clear how strong the evidence is that future self-identification indeed helps goal-directed behaviors and in what way (i.e., as a mediator or moderator). What kind of evidence can be brought to support the authors line of reasoning and how strong is this evidence (i.e., how ‘necessary’ is the variable future self-identification for the ultimate criterion)? Why didn’t the authors directly investigate the effect of the (interaction between the) intervention and personality on whether the participants set (more future-oriented/better) goals and/or whether they adhered to these goals?

*Response: We agree with the Reviewer that these are all interesting questions. However, the aim of the study was to examine the applicability of the situational affordances framework in an intervention context. Therefore, we focused on moderation effects of situations on the expression of personality. We used the proximal outcomes of the FutureU intervention, i.e., future self-identification variables, rather than distal outcomes. Generally, intervention effects are more pronounced immediately after the intervention in the proximal outcomes than in the distal outcomes. Hence, if there would be an interaction effect with personality traits, these would most likely show on the variables representing the proximal outcome of future self-identification. Questions addressing the effectiveness of the intervention and testing of the theoretical intervention model are important, though beyond the scope of this paper*.

7. Interaction effects – that are usually small – are notably difficult to find and replicate, so I wondered about the statistical power of the analyses, especially of the three-way interaction. Can the authors provide a power analysis?
*Response: We agree with the Reviewer that interaction effects are usually small. We ran a power analysis using G*Power. With an effect size of f = 0.143 and* *α error probability of 0.05 the power (1 – β error probability) to detect a three-way interaction is 0.95. We have added this power analysis as a footnote in the analyses section.*

*“Power analysis ran in G*Power showed a power of .95 to detect a three-way interaction, with an f = .14 and an α error probability of 0.05.” (p.14)*

8. As a reader, I prefer to first see a table with all correlations and descriptives (including M, SD, and reliabilities), containing (at least) gender, age, condition, personality variables, and (T1 through T4) criteria variables. In the supplement, this same table is preferably split in two (one for control condition, one for experimental condition). I especially wondered about the relations between the three criteria variables. What does factor-analysis show? Are these indeed independent factors, or might it be useful (and more parsimonious) to conduct the analyses on a higher-order (general) factor?

*Response: We have added a Table with the correlations and descriptives of personality and the outcome variables for the complete sample and moved the Table with descriptives per condition to the supporting materials. The descriptives of gender, age and condition are reported for the total sample in the method section under the heading “Participants”. The reliabilities of the separate instruments are also reported in the method section under the heading “Measurements”.*

*Regarding the criteria variables, factor analyses suggest a two-factor structure at each timepoint representing the subscales vividness and relatedness. The item representing valence loads on the factor of vividness. It may be possible to have a hierarchical model where the items load on two latent factors representing vividness and relatedness that, in turn, load on one latent factor representing future self-identification. However, this hierarchical model would add parameters to be estimated, making the models more complex rather than more parsimonious.*

9. The effect of the intervention on vividness from T1 to T2 seems especially due to the high scores on vividness in T1 in the control group, so it is questionable whether this is actually due to the intervention, because there may be a ceiling effect for the level of vividness that participants experience. The sentence in the discussion that ‘The main effects … indicated that … FutureU … may be a promising intervention for stimulating future self-identification’ is too strong given the (lack of) evidence.

*Response: We do not agree with the Reviewer that there may be a ceiling effect for the level of vividness for participants in the control condition who scored higher on vividness at T1 than participants in the intervention condition. Vividness is measured on a 7-point scale. At T1, participants in the control condition have an average score of 3.50, indicating that they can increase another 3.50 points. In addition, the calculated effect sizes concern the change in scores (see formula p. 14), that is, the change per condition participants show, e.g., from T1 to T2. Differences at T1 between conditions do not affect this effect size calculation.*

*We do agree with the Reviewer though that we cannot draw firm conclusions about the effectiveness of the FutureU intervention and therefore mention that the intervention “may be promising”, that there was a “small positive effect”, explicitly emphasizing that there were no effects on valence and relatedness, and stress “the importance to further optimize the intervention” (p. 22).*

10. In general, my suggestion is to rewrite the manuscript by focusing on the intervention as a moderator of the personality – future self-identification relation (instead of personality as a moderator). This may help putting more in the foreground the important finding that Conscientiousness and Extraversion are (strong) personality predictors of future self-identification, something that – as far as I know – has not been studied before. I realize that this doesn’t strictly align with the preregistration, but I don’t see a problem with clarifying this in the manuscript (e.g., using a footnote).

*Response: We thank the Reviewer for the suggestion to focus the manuscript on the intervention as a moderator. We have adjusted the manuscript (including the title) to emphasize that we examined intervention situations as a moderator of the relation between personality traits and future self-identification. We now also explicitly mention in ‘The present study’ that we included the direct effects of the intervention and personality traits on future self-identification. However, we do not address these direct effects in depth.*

*For example, “To this end, we described the FutureU intervention in terms of intervention situations and corresponding affordances and examined whether the intervention situation (compared to the control condition) moderated the relation between personality traits and intervention effects.” (p. 8)*

*“Interactions of personality traits with intervention situations on the three future self-identification outcome measures (i.e., vividness, valence, and relatedness) were also analyzed with linear mixed regression models for each outcome separately.” (p. 13)*

*“(…) Intervention situations moderated the relation between Extraversion and intervention effects on for valence towards the future self.” (p. 17)*

*“Openness to Experience did not interact with intervention situations either.” (p. 23)*

*“The proposed framework showed potential for systematically understanding (differences in) intervention effects through intervention situations affording the expression of certain personality traits.” (p. 26)*

*“Furthermore, we expected a direct, positive effect of the FutureU intervention on future self-identification given that it aims to increase people’s identification with their future self. We had no specific hypotheses regarding direct effects of personality traits on future self-identification.” (p. 9)*

Good luck with the manuscript!

*Thank you!*

**Reviewer #2:**

1. My first remark refers to the interpretation of results. In the preliminary results, the authors found an intervention effect for vividness but not for valence or relatedness. However, this finding was not discussed (apart from further optimizing the intervention). How could the intervention be optimized? Could one assume that people simply have a clearer image of themselves (i.e., more vividness) due to the presentation of their older avatar? If so, which adjustments would be possible to also address valence and relatedness?

*Response: As the focus of the present study was on the situational affordances framework in which we examined moderation of personality effects by intervention situations, the analyses that were conducted were specifically chosen to answer that (preregistered) research question. Drawing firm conclusions about the effectiveness of the intervention and methods to optimize it would require more in depth, person-centered analyses to understand what exactly is going on. As stated in the paper, the findings “demonstrate the need to study intervention effects on individual level” (p. 22). That said, we have added the following to the discussion section that the visualization of the future self may be able to increase vividness but might not be enough to increase valence and relatedness:*

*“Possibly, seeing a rendered version of one’s future self is enough to increase vividness but may not affect feelings or perceived connection with it despite moments of interaction. Hence, the findings highlight the importance to further optimize the intervention and, at the same time, demonstrate the need to study intervention effects on the individual level.”* *(p. 22)*

2. The primary results revealed that there was only one significant interaction (for Extraversion), in contrast to their assumptions based on the framework. In the discussion, the authors mentioned that the lack of moderation could be explained by the control condition, which might have afforded certain traits as well. How could the intervention be modified so that the control condition does not afford the same trait? What would be the next steps?
*Response: We believe that the first next step needs to be a more rigorous experimental test of the situational affordances framework in an intervention context. As we mention in the discussion section under “Limitations”, the intervention consisted of multiple situations which does not allow for testing specific trait activation propositions in isolation. An experiment comparing a focused intervention (e.g., consisting of a single intervention component, such as goal-setting) with a passive control condition (i.e., encountering no intervention situations) would be an important step to identify specific trait activation. We have added this example in the conclusion section.*

*“Before conclusions either confirming or rejecting situational affordances as a framework in an intervention context can be drawn, future research specifically designed for this aim should further test this. This could, for instance, be achieved by comparing a single component intervention with a control condition without intervention situations.” (p. 26)*

3. Was a correction for multiple testing applied? If not, could the authors explain the rationale behind it?

*Response: We did not correct for multiple testing. Instead, we opted for testing the robustness of our results by conducting sensitivity analyses and comparing the results. Given that moderation of Extraversion was apparent in the results of the full sample as well as in the results of the sensitivity sample, we interpret this finding with more confidence. In contrast, the moderation of Agreeableness was only significant in the sensitivity sample (despite a ‘trend towards significance’ (p = .056) in the full sample) and therefore we stressed the importance of replicating this finding (“…the fact that it only emerged for a subsample of participants, it needs to be replicated before drawing conclusions” p. 25).*

4. Furthermore, I was thinking about alternative explanations for the results. Situational affordances are categorized as environmental variables that can clearly influence behavior, but person variables (like need or goals) also play an important role. Maybe it is not only about situational affordances but more about person situation fit (cf. Rauthmann, 2021)? And what about individual situation perception? Shouldn’t situation characteristics be included in the future to better understand whether the intervention actually affords the expression of certain traits? I think this could be discussed.

*Response: We agree with the Reviewer that the match between person and situation is of importance. In the introduction we formulate that for example as “the match between these situations and traits” (p. 4) and “eliciting different responses by individuals as a function of their trait levels in which some situations of an intervention may ‘fit’ people and other situations may not” (p. 5).*

*We also agree with the Reviewer that the same situation can be perceived differently by individuals and that perception of a situation may also be related to personality traits. However, given that we do not have data on how participants in our study perceived different situations, elaborating on this matter would remain entirely speculative and draw away from the aim of the present paper which was to test the situational affordances framework in an intervention context. Therefore, we now mention this in the discussion section as a direction for future research.*

*“Additionally, the affordances provided by situations to express certain personality traits is based on theory. However, how a situation is perceived could differ between individuals and may, therefore, also afford the expression of different personality traits for different people. For future research, it would be interesting to study situations and afforded expressions of traits in isolation as well as whether people perceive affordances of situations for the expressions of traits in the same way and in line with theoretical considerations.” (p. 25)*

5. The authors state that open data were available and provide a link to an online platform, OSF. However, data were not openly available. The link to the data and code didn’t work. I guess this was due to a typo (“il” in the link instead of “io”, 295): However, even changing this typo does not solve the issue. Please add a View-only link in a potential revision of the article.

*Response: We apologize. We have made the project of the present paper on OSF publicly available and have adjusted the typo.*

6. Concerning the figures, I was wondered if it would be possible to display the three groups (six trajectories) into one panel/graph? This way it would be easier to directly recognize the effects and compare them across groups. I would also recommend using confidence intervals around the point estimates (if possible). Finally, I would suggest mentioning the range of the y-axis in the note of the figure as it is truncated.

*Response: We understand the point the Reviewer makes. However, displaying the six trajectories into one graph would be confusing for the results concerning Agreeableness (Figure 4) as the three trajectories of the intervention condition are highly similar from T1-T3 (see Figure below). For consistency, we display the three groups in three graphs for both moderation effects. We have added the confidence intervals and a note to the Figures that the range of the y-axis is truncated.*

7. Generally, I think the limitations section could be more specific. The authors said, for instance, that the sample size was rather small for personality research (469). What would be a possible consequence of this? Why should it be a much larger sample for personality, and is the effect likely overestimated or underestimated?

*Response: Larger sample sizes make it statistically easier to obtain significant results, due to smaller standard errors. Given the modest sample size used in the present study, the results are more likely to be underestimated than overestimated.*

*We now explicitly mention this in the limitations section.*

*“As a consequence, significant effects are less likely to show and effect size estimates are less precise.” (p. 26)*

Additional comments:

- The order of authors differs on the manuscript and on the completed form. Perhaps the authors would like to check this again.

*Response: We thank the Reviewer for pointing this out and have checked this.*

- For vividness, the authors mentioned that items were answered on a 7-point Likert scale (228). Please consider this as a suggestion (I’m aware that many people use this wording), but I would recommend writing rating scale instead, as technically a Likert scale is a combination of items (cf. Likert, 1932).

*Response: We adjusted this as suggested.*


References:

Likert, R. (1932). A technique for the measurement of attitudes. Archives of Psychology, 22 140, 55.

Rauthmann, J. F. (2021). Capturing interactions, correlations, fits, and transactions: A Person-Environment Relations Model. In The Handbook of Personality Dynamics and Processes (pp. 427–522). Elsevier. <https://doi.org/10.1016/B978-0-12-813995-0.00018-2>
